# Iron loading and disease surveillance.

**DOI:** 10.3201/eid0701.010131

**Published:** 2001

**Authors:** M Reyes, G Imperatore


The *catP* gene has been found on various bacterial chromosomes and conjugative plasmids as part of the transposable element Tn*4451*. This transposon is derivative of the Tn*6338* that contains six genes, the largest of which, *TnpX,* is required for the excision of the transposon in both *Escherichia coli* and *C. perfringens* (6). The Tn*4451* derivative that lacked the functional *TnpX* gene was completely stable in both organisms because it had lost mobility as a result of these internal deletions (6). The finding of *catP* in *N. meningitidis* within such a truncated immobile transposon (1) and the possibility of transfer of this type of resistance among highly transformable organisms such as *Neisseria* spp. are of great concern. Were the transposon to become a stable part of the meningococcal genome, it could potentially be easily exchanged. Interspecies recombination between antibiotic-resistant genes of *N. meningitidis* and commensal *Neisseria* spp. has occurred in penicillin- and sulfonamide-resistant meningococci (7-10). A similar occurrence may be possible for *N. meningitidis* chloramphenicol resistance in Africa or other continents where this antibiotic is routinely used for treatment of patients with meningococcal diseases. Studies have not yet demonstrated the clinical significance of chloramphenicol resistance caused by the *catP* gene in meningococci. However, it is possible that, in developing countries, patients whose illness does not respond to antimicrobial agents may not be detected, or their isolates may not be obtained. Screening a selection of isolates for *catP* may allow early detection of chloramphenicol-resistant strains. Since detection of increasing chloramphenicol resistance could change recommendations for antimicrobial-drug therapy, surveillance for antimicrobial-drug resistance should be encouraged.

### Acknowledgment

We gratefully acknowledge Jasmine M. Mohammed for performing chloramphenicol and penicillin MICs.

**Maria-Lucia C. Tondella, Nancy E. Rosenstein, Leonard W. Mayer, Fred C. Tenover, Sheila A. Stocker, Mike W. Reeves, and Tanja Popovic**
Centers for Disease Control and Prevention, Atlanta, Georgia, USA

### References

1. Galimand M, Gerbaud G, Guibourdenche M, Riou JY, Courvalin P. High-level chloramphenicol resistance in *Neisseria meningitidis*. N Engl J Med 1998;339:868-74.
2. World Health Organization. Control of epidemic meningococcal disease. WHO Practical Guidelines. 2nd ed. Geneva: World Health Organization; 1998. p.1-84 (WHO/EMC/BAC/98.3).
3. Selander RK, Caugant DA, Ochman H, Musser JM, Gilmour MN, Whittam TS. Methods of multilocus enzyme electrophoresis for bacterial population genetics and systematics. Appl Environ Microbiol 1986;51:873-84.
4. Caugant DA. Population genetics and molecular epidemiology of *Neisseria meningitidis*. APMIS 1998;106:505-25.
5. National Committee for Clinical Laboratory Standards. Methods for dilution antimicrobial susceptibility tests for bacteria that grow aerobically. 5th edition: approved standard, M7-A5. Wayne, PA: National Committee for Clinical Laboratory Standards; 2000.
6. Bannam TL, Crellin PK, Rood JI. Molecular genetics of the chloramphenicol-resistance transposon Tn4451 from *Clostridium perfringens*: the *TnpX* site-specific recombinase excises a circular transposon molecule. Mol Microbiol 1995; 16:536-51.
7. Spratt BG, Zhang QY, Jones DM, Hutchison A, Brannigan JA, Dowson CG. Recruitment of a penicillin-binding protein gene from *Neisseria flavescens* during the emergence of penicillin resistance in *Neisseria meningitidis.* Proc Natl Acad Sci U S A 1989;86:8988-92.
8. Sáez-Nieto JA, Lujan R, Martinez-Soares JV, Berron S, Vazquez JA, Vinas M, et al. *Neisseria lactamica* and *Neisseria polysaccharea* as possible sources of meningococcal beta-lactam resistance by genetic transformation. Antimicrob Agents Chemother 1990;34:2269-72.
9. Bowler LD, Zhang QY, Riou JY, Spratt BG. Interspecies recombination between the *penA* genes of *Neisseria meningitidis* and commensal *Neisseria* species during the emergence of penicillin resistance in *N. meningitidis*. J Bacteriol 1994;176:333-7.
10. Oppenheim BA. Antibiotic resistance in *Neisseria meningitidis*. Clin Infect Dis 1997;24(suppl 1):S98-S101.

### Iron Loading and Disease Surveillance

**To the Editor:** We read with interest the article by E. D. Weinberg entitled “Iron Loading and Disease Surveillance” (1). Dr. Weinberg proposes routine population screening of iron values by serum ferritin and transferrin saturation tests. Such screening could provide valuable information for epidemiologic, diagnostic, prophylactic, and therapeutic studies of emerging infectious diseases. However, population screening for hereditary hemochromatosis, the example Dr. Weinberg uses to illustrate his proposal, should await additional data (2-4). At this time, it is not known how many people with genetic risk or biochemical evidence of iron overload will actually become ill. Therefore, the benefits of screening cannot be weighed against the risks of unnecessary treatment. Moreover, standardized, reliable methods for measuring and diagnosing iron overload are not available.

Without additional data, population screening can actually be detrimental to those at risk for disease. Persons with hereditary hemochromatosis may face discrimination, including difficulties in acquiring health, life, or disability insurance. Already, current blood safety policy makes it difficult for them to donate blood, even though blood donation is unlikely to have negative consequences. In addition, the costs of screening for hemochromatosis are not routinely covered by medical insurance nor has the cost-effectiveness of screening been determined. If routine screening is adopted, tracking of persons who test positive must be developed to ensure that appropriate and continuing follow-up is provided and patient confidentiality is preserved.

The Centers for Disease Control and Prevention recommends testing for persons who have either a close relative with hemochromatosis or who themselves experience the unexplained symptoms compatible with the disease (severe weakness or fatigue; unexplained joint or abdominal pain) or its later complications (liver disease, diabetes, or heart problems; impotence; infertility; loss of menstrual periods) (2,5). Testing to exclude other causes of these medical problems should also be performed. Persons with elevated iron or liver function measures should be monitored by their health-care provider.

**Michele Reyes and Giuseppina Imperatore**
Centers for Disease Control and Prevention, Atlanta, Georgia, USA

### References

1. Weinberg ED. Iron loading and disease surveillance. Emerg Infect Dis 1999;5:346-52.
2. Cogswell ME, Mc Donnell SM, Khoury MJ, Franks AL, Burke W, Brittenham G. Iron overload, public health, and genetics: evaluating the evidence for hemochromatosis screening. Ann Intern Med 1998;129(Suppl 11);971-9.
3. Cogswell ME, Burke W, McDonnell SM, Franks AL. Screening for hemochromatosis. A public health perspective. Am J Prev Med 1999;16:134-40.
4. EASL International Consensus Conference on Haemochromatosis. J Hepatol 2000;33:485-504.
5. Witte DL, Crosby WH, Edwards CQ, Fairbanks VF, Mitros FA. Practice guideline development task force of the College of American Pathologists. Hereditary hemochromatosis. Clin Chim Acta 1996;245:139-200.

### Reply to Dr. Reyes

**To the Editor:** The article noted that nearly 50 microbial genera contain strains that are more pathogenic in iron-loaded than in normal hosts. The article proposed "routine screening of populations exposed to certain diseases" but not routine screening of populations at large. A few examples of current interest include atherosclerosis (*Coxiella* and *Chlamydia*), septicemia (*Capnocytophaga*), Whipple's disease (*Tropheryma*), tuberculosis (*Mycobacterium*), gastric ulcers (*Helicobacter*), hepatitis (hepatitis C), and AIDS (opportunistic pathogens).

Of course, the tissue or cell localization of iron and the possible pathogen must be considered. For instance, *Legionella* multiplies in iron-loaded alveolar macrophages but not in plasma. Thus, it would be expected that persons with untreated hemochromatosis with minimal macrophage iron but with high plasma iron would not be at risk for Legionnaires' pneumonia.

**Eugene D. Weinberg**
Indiana University, Bloomington, Indiana

